# A Robust Balance-Control Framework for the Terrain-Blind Bipedal Walking of a Humanoid Robot on Unknown and Uneven Terrain

**DOI:** 10.3390/s19194194

**Published:** 2019-09-27

**Authors:** Hyun-Min Joe, Jun-Ho Oh

**Affiliations:** 1 Department of Artificial Intelligence Machinery, Korea Institute of Machinery & Materials, 156 Gajeongbuk-ro, Yuseong-gu, Daejeon 34103, Korea; hyunmin.joe@gmail.com; 2 HUBO LAB, Department of Mechanical Engineering, Korea Advanced Institute of Science and Technology, 291 Daehak-ro, Yuseong-gu, Daejeon 34141, Korea

**Keywords:** balance control, biped walking, capture point, DRC-HUBO+, humanoid robot, terrain-blind walking, zero-moment point

## Abstract

Research on a terrain-blind walking control that can walk stably on unknown and uneven terrain is an important research field for humanoid robots to achieve human-level walking abilities, and it is still a field that needs much improvement. This paper describes the design, implementation, and experimental results of a robust balance-control framework for the stable walking of a humanoid robot on unknown and uneven terrain. For robust balance-control against disturbances caused by uneven terrain, we propose a framework that combines a capture-point controller that modifies the control reference, and a balance controller that follows its control references in a cascading structure. The capture-point controller adjusts a zero-moment point reference to stabilize the perturbed capture-point from the disturbance, and the adjusted zero-moment point reference is utilized as a control reference for the balance controller, comprised of zero-moment point, leg length, and foot orientation controllers. By adjusting the zero-moment point reference according to the disturbance, our zero-moment point controller guarantees robust zero-moment point control performance in uneven terrain, unlike previous zero-moment point controllers. In addition, for fast posture stabilization in uneven terrain, we applied a proportional-derivative admittance controller to the leg length and foot orientation controllers to rapidly adapt these parts of the robot to uneven terrain without vibration. Furthermore, to activate position or force control depending on the gait phase of a robot, we applied gain scheduling to the leg length and foot orientation controllers, which simplifies their implementation. The effectiveness of the proposed control framework was verified by stable walking performance on various uneven terrains, such as slopes, stone fields, and lawns.

## 1. Introduction

Considering the eventuality of a future accident, such as that of the Fukushima power plant nuclear accident [1], the DARPA Robotics Challenge was held to test robots performing tasks that would replace human ones in disaster environments resembling the Fukushima accident [2]. Environments where humans act, including power plants, are designed with consideration for human morphology. This fact implies that humanoid robots are suitable candidates for carrying out tasks in such environments and employing human-made objects, and most of the teams in the challenge used humanoid robots. However, many of these robots were limited by unstable walking, especially when ground slope and height variation appeared during the tasks.

Although many robots used vision and LiDAR sensors for the perception of uneven terrain, inherent errors of the sensors and the measurements the environment caused terrain sensing errors. In order to ensure stable locomotion in the presence of uncertain information regarding the ground, a robot should have the ability of balance control to overcome the ground information error. In addition, the robustness of balance control is more important than the accuracy of ground terrain sensing for environments composed of rough local terrain smaller than the foot, such as in a gravel field. Thus, for a humanoid robot to accomplish a mission in real-world environments, a robust, terrain-blind walking control is required to prevent falls on unknown and uneven terrain. The aforementioned observations show the necessity of terrain-blind walking control research, which can guarantee stable locomotion without terrain information to account for uncertain ground information and improve balance-control abilities. 

The most basic and important balance-control method for a biped humanoid robot with a finite sized foot is maintaining the zero-moment point [3] within the support polygon. Nagasaka [4] proposed a method of stabilizing the zero-moment point (ZMP) using the acceleration of the humanoid torso based on the cart-table model. Likewise, Choi et al. [5] proposed a method to balance a humanoid robot through ZMP error feedback. In another approach, Kim et al. [6] implemented the stable walking of the humanoid robot HUBO by designing an observer-based state feedback controller to regulate the ZMP by estimating the state of the robot from the ZMP. Martinez at al. proposed a method to more accurately obtain a ZMP control model, improving ZMP control performance [7]. These methods are effective on flat ground, but their performance is not guaranteed on uneven terrain, thus imposing the necessity of robust ZMP controllers for unknown, uneven terrain. 

In addition to the ZMP, it is important to maintain posture along the walking pattern during biped walking. To maintain posture on unknown uneven terrain, the foot position and orientation should be controlled, and contact force control is commonly used for adapting the foot to unknown uneven terrain. For instance, Ott et al. [8] proposed a contact force distribution and control method inspired by the grasping method. Furthermore, quadratic-programming based balance controllers have been proposed that calculate the optimal joint torque input, considering constraints [9,10,11]. Among them, Feng et al. [8] developed a quadratic-programing-based balance controller to simultaneously optimize the joint acceleration, joint torque, and contact force. These controllers are implemented on joint-torque-controlled humanoid robots (e.g., TORO [12], Atlas [13], and Valkyrie [14]) and stabilize the robots’ postures on unknown uneven terrain. However, these controllers cannot be used in position-controlled humanoid robots (e.g., DRC-HUBO+ [15], HRP-4 [16], and JAXON [17]). 

Position-controlled robots rely on impedance or admittance control for contact force control. Lim et al. [18] applied a position-based impedance control to reduce the impact forces generated between the landing foot and the unknown, uneven terrain. Similarly, Kim et al. [19] applied admittance control by modeling the leg as a spring–damper system, and Kajita et al. [20] proposed a linear inverted pendulum tracking controller and a damping controller, which is a kind of admittance control, to regulate posture. However, these position-based contact-force control methods do not show comparable performance relative to their torque-based counterparts [9,10,11]. 

Considering the limitations of the studies presented above, in this paper, we propose a robust balance control framework to improve the terrain-blind walking performance of a position-controlled humanoid robot. To build a control frame that is robust against disturbances from uneven ground and enables fast posture stabilization on challenging terrain that conventional position-controlled humanoid robots fail to navigate, we synthesized a capture-point controller [21,22,23] and a balance controller in a cascaded structure. We applied a capture-point controller, which proved robust to disturbances, as a control reference generator for the balance control framework. The disturbed state of the robot is reflected in the capture point, and the capture-point controller adjusts the ZMP reference to stabilize the perturbed capture point. The adjusted ZMP reference is used as a control reference in the balance controller.

The balance controller is comprised of a ZMP controller to stabilize the motion of the center of mass (CoM) and a posture controller to stabilize the posture of the robot. Our ZMP controller is designed to control the ZMP in uneven terrain by receiving a ZMP reference that considers disturbances from capture-point control. In addition to stabilizing the CoM through ZMP control, leg length and foot orientation controllers act as posture controllers that adapt the leg length and foot orientation to the shape of the terrain to maintain the posture of the robot. Then, for faster posture stabilization in challenging terrain, we applied a proportional-derivative (PD) admittance controller to each posture controller to enable fast reference tracking without oscillation. Furthermore, we applied the gain scheduling method for the convenience of implementation when the leg length and foot orientation controllers were applied to the robot. To test the performance and effectiveness of the proposed control framework, we implemented it on the DRC-HUBO+ humanoid, whose arms and head were replaced by two dumbbells with the same weight. Then, we conducted various experiments under different ground conditions. Videos from the experiments can be seen in [24].

The rest of this paper is organized as follows. The overview of robot system and proposed balance control framework is described in Section 2. In Section 3, we describe the ZMP controller for stabilizing the horizontal CoM motion on tilted ground using capture-point control. Section 4 presents the design of the leg length and foot orientation controllers for adaptation to uneven terrain. In Section 5, experimental results of balance maintenance on different uneven terrain conditions are reported. In Section 6, we draw conclusions based on the experimental results.

## 2. Control Framework and Robot System

### 2.1. Balance Control Framework

Bipedal walking control systems are traditionally composed of a walking pattern that generates the walking motion of the robot and a balance controller that stabilizes the walking pattern from disturbance [25]. In an ideal environment and conditions, the robot can walk with only a walking pattern, but in real environments, various disturbances act on the robot, so a balance controller must be designed to stabilize the walking pattern through sensor feedback.

**Figure 1:**
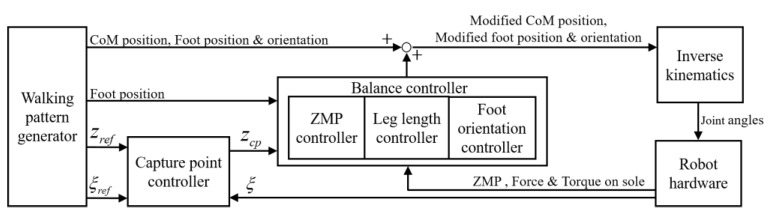
Overview of the information flow in proposed balance control framework.

An overview of the proposed balance control framework is shown in Figure 1. The walking pattern generator uses preview control [26] to create a CoM reference. The walking pattern also generates a ZMP reference ($z_{ref}$), capture-point reference ($\xi_{ref}$), desired posture reference, foot position reference, and foot orientation reference. To design a robust balance controller, we applied capture-point control, which proved robust to disturbance, to the balance control framework. In the capture-point controller, a $z_{cp}$ reference is generated to maintain the balance of the robot. The generated $z_{cp}$ is used as a reference for a balance controller composed of ZMP, leg length, and foot orientation controllers. Because the CoM behavior represents the stability of the bipedal walking robot system, the ZMP controller is applied for CoM stabilization. The ZMP controller receives the $z_{cp}$ input generated through the capture-point control and controls the ZMP tracking. Because CoM stabilization alone is insufficient for maintaining the walking pattern posture in uneven terrain, leg length and foot orientation controllers are added to the balance controller to maintain a desired posture. The following sections describe, in detail, each component of the balance controller. Finally, the control inputs from the balance controller and the walking pattern are converted into joint angles ($\theta_{ref}$) by inverse kinematics and each joint ane controller of the robot tracks the generated joint angles.

### 2.2. Robot System

**Figure 2:**
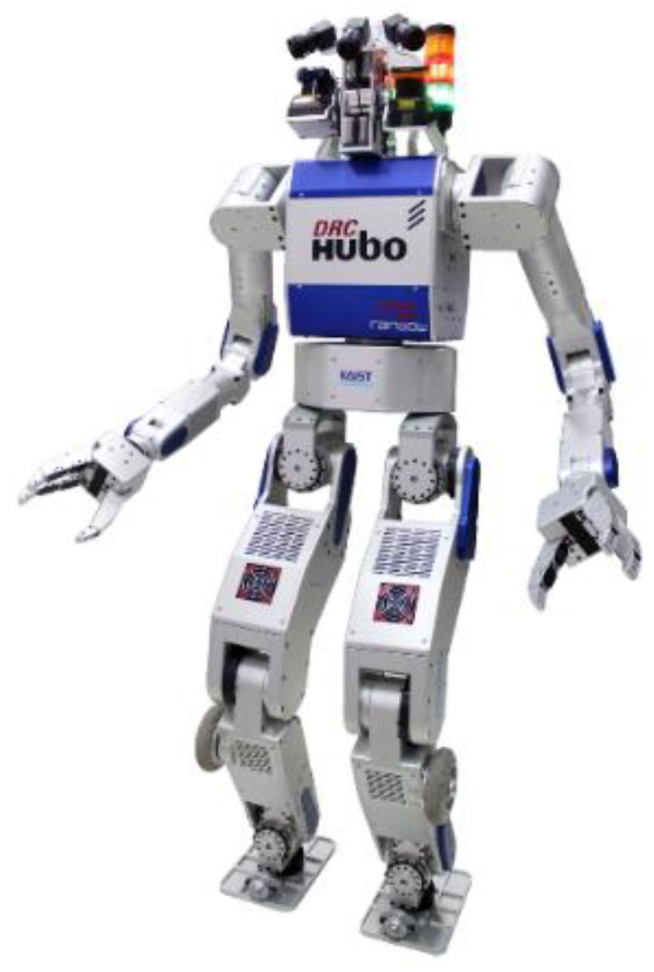
Humanoid Robot DRC-HUBO+.

Figure 2 shows the humanoid robot DRC-HUBO+. DRC-HUBO+, a disaster rescue robot developed by HUBO LAB (a mechanical engineering department of the Korea Advanced Institute of Science and Technology) was designed to participate in the DARPA Robotics challenge in 2015. Rainbow Robotics currently produces and sells it with distributorship. In the actual robot experiment, we used a modified DRC-HUBO+. Because the humanoid robot, which is a floating base system, tends to break down when it falls down, and the proposed balance controller does not use arms and heads, DRC-HUBO+’s head and arm were replaced by dumbbells of the same weight. Considering the points mentioned above, when developing a bipedal walking controller of a humanoid robot, it is often the case that there is no arm or head. Because the immobile arm and head connected to the torso were modeled as a single rigid body, there was no performance difference in terms of control after the robot was modified.

The DRC-HUBO+ is designed with high structural stiffness and high joint torque capacity. A harmonic drive was used to output high torque to each joint, and the frame of the robot was designed in the form of an exoskeleton for high rigidity with respect to weight. Table 1 shows the basic hardware specifications. The robot has a height of about 1.7 m and weighs 80 kg, including two batteries with a weight of 4 kg each. The joint angle references generated at a frequency of 200 Hz in the embedded PC are transmitted to each joint controller via CAN (Controller Area Network) communication, and each joint controller performs joint angle control of the PD gain at a frequency of 1 kHz. Force/torque (F/T) sensors (6-axis strain gauge sensors developed by KAIST HUBO LAB) and a gyro sensor (a DSP-1760 fiber optic gyro, KVH), attached to both the ankle and pelvis, are used for sensor feedback control. The F/T sensor is used to calculate the ZMP for ZMP feedback control; a description of the ZMP calculation is given in [25]. ZMP feedback control is described in Section 3. F/T feedback is described in Section 4. The gyro sensor is used to calculate the CoM position for capture-point feedback [21,22]. More detailed hardware specifications and system configurations are available in [15].

## 3. Stabilization of the CoM

The CoM dynamics are reflected to the ZMP through the interaction between the foot and floor, and the motion of CoM can be stabilized indirectly through the ZMP control [6,27]. The motion of the CoM is stabilized by designing the ZMP controller to follow the desired ZMP reference. Therefore, to stabilize the horizontal CoM motion, we propose a more robust ZMP controller by combining capture-point control with a previously developed ZMP controller [6,27]. Unlike the previous ZMP controller, this enhanced ZMP controller is capable of ZMP control on flat as well as tilted ground.

### 3.1. Previous ZMP Control

Kim et al. [27] added a damping element to the ZMP control model of the biped humanoid robot KHR-2 [6] to consider the damping factor of the six-legged robot, and implemented stable ZMP control of the six-legged robot. This ZMP controller can be applied to various types of legged robots if ZMP can be measured. In this paper, the ZMP control model of [27] is applied, considering the damping factor of DRC-HUBO+, and it is extended to a more robust ZMP controller by combining the ZMP controller of [27] and the capture-point control of [21,22,23].

**Figure 3:**
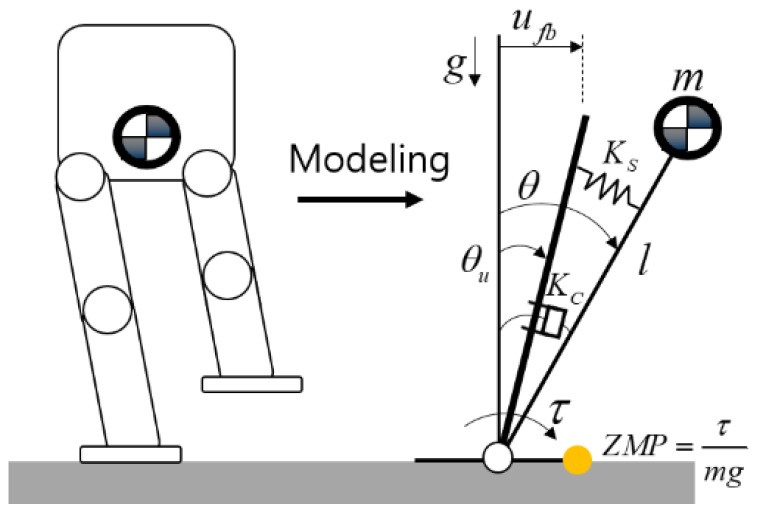
Inverted pendulum model with spring and damper.

In [26], the robot is modeled as the inverted pendulum with spring and damper (IPSD) shown in Figure 3. We describe the model with respect to the frontal plane (y-axis direction) for convenience, and the same process is applied to the sagittal plane (x-axis direction). Equations (1)–(5) are derived from [27]. The equation of motion of the linearized dynamic model of the inverted pendulum is given by (1), where $K_{c}$ is the damping coefficient, $K_{s}$ is the spring constant, g is the gravitational acceleration, m is the point mass, l is the inverted pendulum length, θ is the inclination angle, $\theta_{u}$ is the command angle, and $u_{fb}$ is the horizontal displacement equal to $l\theta_{u}$.

(1) $$ml^{2}\ddot{\theta }=mgl\theta -\tau$$

where $\tau =K_{s}\left(\theta -\theta_{u}\right)+K_{c}\dot{\theta }$.

From the equation of motion, the state-space equations with input and output being horizontal displacement $u_{fb}$ and ZMP *z* are derived as

(2) $$\dot{x}=Ax+Bu_{fb},$$

where $x=\left[\begin{array}{c} \theta \\ \dot{\theta } \end{array}\right]$, $A=\left[\begin{array}{cc} 0 & 1\\ \frac{g}{l}-\frac{K_{s}}{ml^{2}} & -\frac{K_{c}}{ml^{2}} \end{array}\right],B=\left[\begin{array}{c} 0\\ \frac{K_{s}}{ml^{3}} \end{array}\right]$,

(3) $$z=Cx+Du_{fb},$$

where $C=\left[\begin{array}{cc} \frac{K_{s}}{mg} & \frac{K_{c}}{mg} \end{array}\right],D=\left[\frac{K_{s}}{mgl}\right]$.

The state observer is designed to estimate state x in Equation (2), which can be modified to an observed state feedback controller as follows:

(4) $$\dot{\hat{x}}=A\hat{x}+\;Bu_{fb}+L\left(z-C\hat{x}-Du_{fb}\right)$$

(5) $$u_{fb}=-K\hat{x}.$$

**Figure 4:**
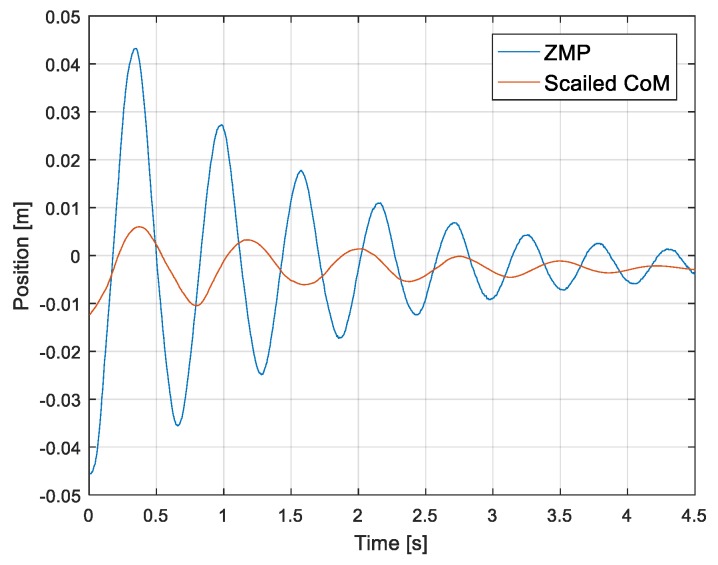
Oscillation of zero-moment point (ZMP) and center of mass (CoM) when a disturbance is applied to the robot. (For improved visualization, the CoM was increased three times).

**Figure 5:**
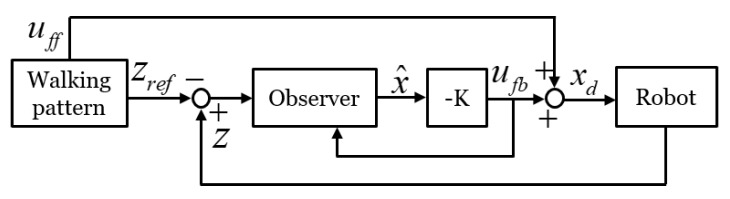
Block diagram of previous ZMP control without capture-point feedback.

Damping coefficient $K_{c}$ and spring constant $K_{s}$ are estimated from the oscillation of the ZMP (Figure 4), which is obtained by applying external disturbance to the standing robot. The external disturbance was applied to the robot by a person pushing the side of the robot by hand for a short moment. The intensity of the external disturbance is such that the ZMP does not reach the edge of the foot sole, and only the joint position controller is applied to the robot, without the balance controller. We estimated the values of $K_{c}$ and $K_{s}$ to be 5774 Nm/rad and 69.375 Nms/rad, respectively. The estimation of these parameters is detailed in [27]. Figure 5 shows the block diagram of the previous ZMP controller [26], where $u_{ff}$ is the feedforward CoM input generated from the walking pattern, $z_{ref}$ is the ZMP reference, and $x_{d}$ is the CoM input to the robot given by the sum of the feedforward and feedback CoM inputs generated from the ZMP control.

The system is lightly damped and non-minimum phase, as shown in Figure 4. The damping of the system should be increased by placing the current pole (−0.732±10.4i) further away from the imaginary axis. Through pole placement, we can determine state feedback gain K and observer gain L. We adjusted the controller poles to reduce the settling time and to prioritize the undershoot minimization. The controller poles were determined as (−4±1.5i) and the poles for the observer were set to be three times faster than the controller poles, based on modern control theory.

**Figure 6:**
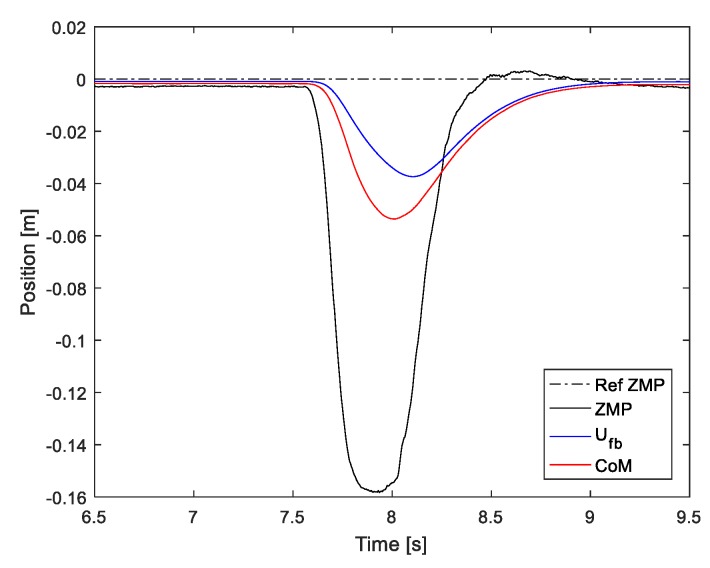
Control input and measured ZMP when the robot is perturbed.

We applied the ZMP controller to the actual robot (DRC-HUBO+) and tested its performance. Figure 6 shows the measured ZMP and control input when a person momentarily pushed the side of the robot. It was confirmed that the measured ZMP is quickly stabilized within 1 s, and the CoM was also stabilized without oscillation (compare with Figure 4), which is the experimental result without the ZMP controller. However, because the ZMP controller estimates the state of the robot from the ZMP, the control performance is not guaranteed when the ground is not flat. In the next section, we describe the proposed ZMP controller that overcomes these drawbacks and compare it with this ZMP controller.

### 3.2. ZMP Controller with Capture-Point Feedback

In [27], the ZMP controller is designed assuming a flat surface for robot walking. As ground inclination was not considered during modeling, the robot becomes unstable on tilted ground. To include ground inclination in the ZMP controller, we utilized capture-point control [21,22,23], whose state reflects the tilting, and the ZMP reference was adjusted through capture-point control. The adjusted ZMP reference is reflected to the observer of the ZMP controller such that the ZMP is stabilized on tilted ground.

In [21,23], capture point ξ is defined by the sum of CoM position x and the scaled CoM velocity $\dot{x}$:

(6) $$\xi =x+\frac{\dot{x}}{\omega },$$

where $\omega =\sqrt{g/h}$ with h being the height from the stance foot to the CoM. Although capture-point error Δξ is ideally zero without disturbance, a disturbance applied to the robot causes the error between the reference ($\xi_{ref}$) and measured (ξ) capture points. To mitigate the capture-point error under disturbance, the following capture-point feedback controller is proposed in [22]:

(7) $$z_{cp}=k_{cp}\underset{\Delta \xi }{\underbrace{\left(\xi -\xi_{ref}\right)}}+z_{ref},$$

where $k_{cp}$ is the capture-point feedback gain and $z_{cp}$ is the adjusted ZMP reference by capture-point control. Note that ZMP $z_{cp}$ for controlling the capture point is the sum of the capture-point error scaled by the gain and the ZMP reference. If $k_{cp}>1$, a ZMP deviation ($k_{cp}\Delta \xi$) greater than the capture-point error is added to the reference, causing the capture-point error to converge in the decreasing direction. The proof of capture-point control is detailed in [22].

**Figure 7:**
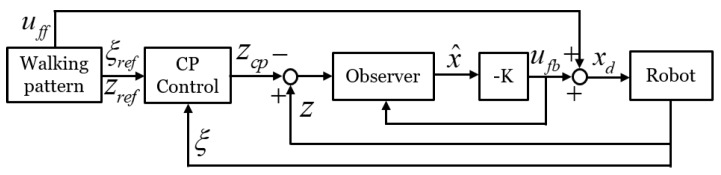
Block diagram of proposed ZMP controller.

Figure 7 shows the block diagram of the proposed ZMP controller, where the disturbance is reflected in the capture-point state, and $z_{cp}$ is used instead of $z_{ref}$ in the observer to reduce the capture-point error. ZMP reference $z_{ref}$ does not contain information of the terrain because it is generated from the walking pattern. In contrast, adjusted reference $z_{cp}$ contains information about the disturbance by reflecting the state of the capture point, hence the robot can be stabilized on tilted ground.

**Figure 8:**
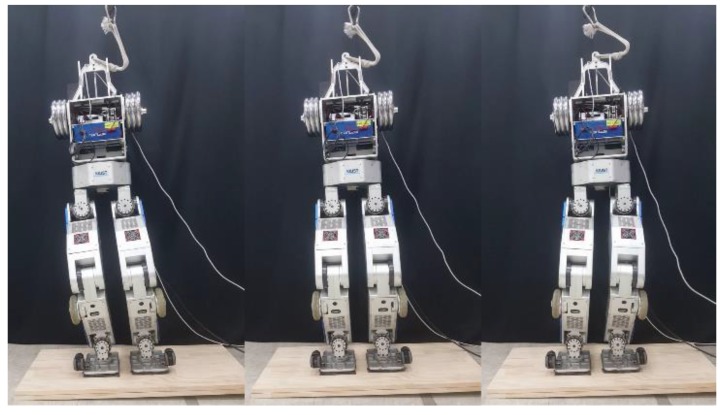
A test of the ZMP controller on an inclined surface. We commanded the CoM trajectory of the robot and measured ZMP data using the proposed and conventional ZMP controllers.

**Figure 9:**
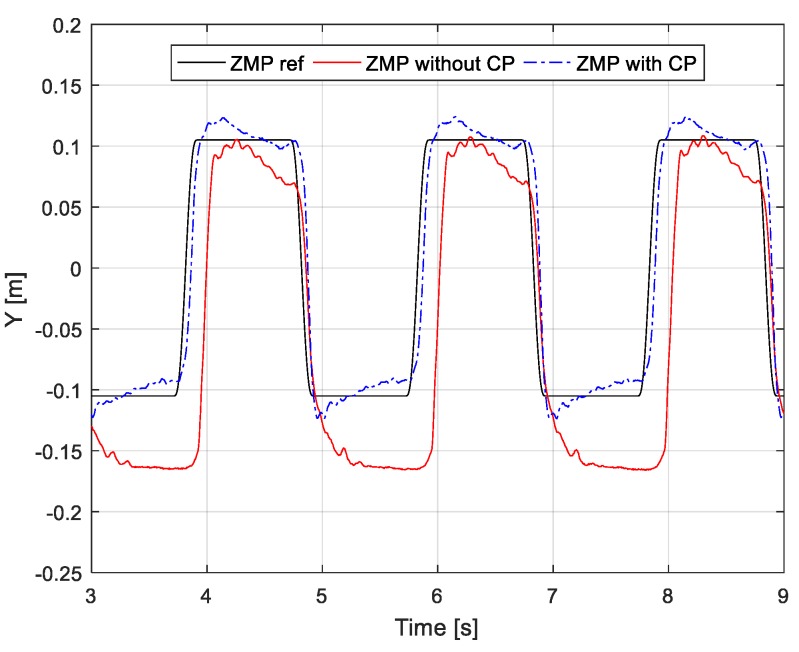
Reference and measured ZMPs with/without capture-point feedback on tilted ground. The reference corresponds to the original walking pattern.

To evaluate the proposed ZMP controller, we compared the proposed and previous controller on a tilted ground. In evaluating the performance of the ZMP controller, not only the ZMP but also the attitude of the robot can be used as a performance evaluation, so we measured the attitude of the robot and the ZMP. As shown in the Figure 8, a horizontal CoM motion from the ZMP reference (Figure 9) was commanded to a robot placed on a tilted surface, and both the ZMP and attitude of the robot (pelvis attitude) were measured by applying each controller. The ZMP is calculated by a Force/Torque sensor mounted on the foot sole, and the attitude of the robot is measured by the IMU sensor mounted on the pelvis. In this paper, we used the preview control [26] to generate the CoM motion, but CoM motion can be generated using various walking pattern generation methods [28,29,30].

**Figure 10:**
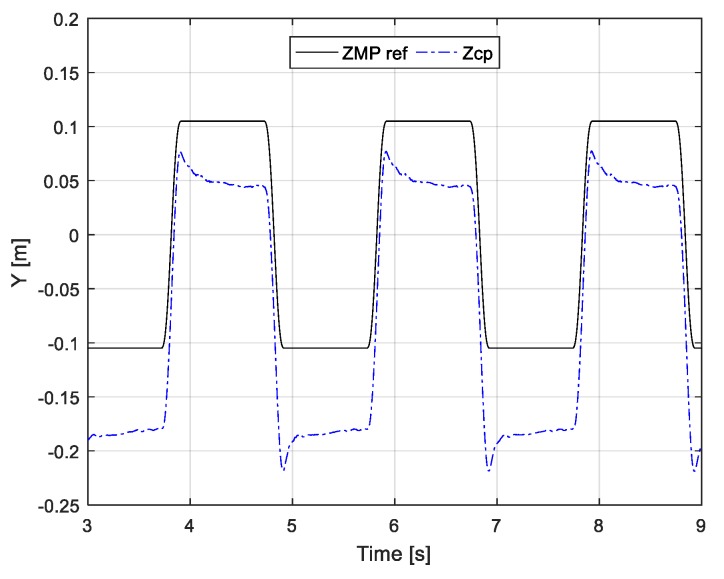
Generated $z_{\mathrm{cp}}$ by capture-point feedback.

The measured ZMP for each ZMP controller on the tilted surface is shown in Figure 9. The previous ZMP controller does not track the reference ZMP well because the disturbance caused by the tilted surface is not considered in the controller design. On the other hand, because the capture-point controller adjusts $z_{cp}$ in order to stabilize the capture point disturbed by the inclination, the proposed ZMP controller provides better ZMP tracking performance than the previous ZMP controller. Figure 10 shows the adjusted $z_{cp}$ and reference ZMP. Because the robot was tilted in the y-direction (frontal plane), $z_{cp}$ was adjusted more in the y-direction than the ZMP reference. In other words, as $z_{cp}$ was adjusted more in an inclined direction than the existing ZMP reference, the ZMP did not move in a tilted direction.

**Figure 11:**
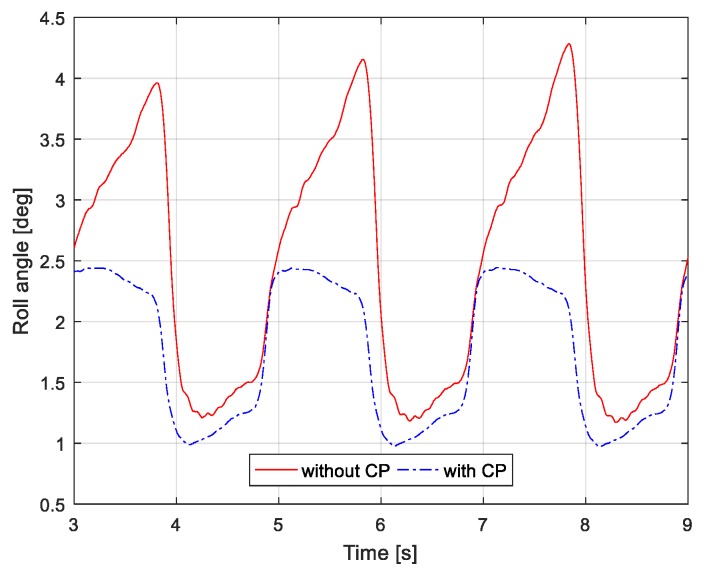
Measured orientation of pelvis with/without capture-point feedback on tilted ground. The orientation was measured from an inertial measurement unit mounted on the robot pelvis.

Figure 11 shows the robot’s attitude measured in the roll direction according to each ZMP controller. When the proposed ZMP controller is applied, the attitude of the robot changes within 0.9°–2.5°, and when the previous ZMP is applied, the attitude of the robot changes within 1° and 4.5°. In the case of the previous ZMP controller, a large postural change of the robot was shown relative to the proposed ZMP controller, because the CoM of the robot was unstable due to unstable ZMP control. On the other hand, in the case of the proposed ZMP controller, because the CoM of the robot is stabilized by stable ZMP control, variation of the posture was relatively small.

When the proposed ZMP was applied, the posture change was relatively small, but there was also a slight posture change. Because the ZMP controller stabilizes the CoM in the horizontal direction, an additional controller for posture stabilization is needed. The leg length and foot orientation controller in the following Section 4 are designed for posture stabilization.

## 4. Stabilization of Posture

In Section 3, the ZMP controller design for the horizontal stabilization of the CoM is provided. The proposed ZMP controller showed satisfactory ZMP control performance on inclined ground through stabilization of the CoM in the horizontal direction. However, it was confirmed that the posture could not be maintained at 0°, which is the desired posture. ZMP control and posture control are required for stabilizing the walking pattern on unknown and uneven terrain.

In the case of flat ground, it is possible to achieve stable walking with only a ZMP controller. However, in the case of uneven terrain, an additional posture controller is needed for adapting to the unknown slope of the floor and the unknown difference in height to maintain the desired posture. Because the CoM trajectory is generated under the assumption that the ground is flat, if the ground is not flat, the robot cannot move according to the desired walking pattern. This causes the instability of walking. Thus, to stabilize the posture, we additionally designed a leg-length controller and a foot orientation controller. In this paper, the aim of the posture control is to maintain the upright posture of the robot. The leg-length control corresponding to the height difference of the ground and the foot orientation control corresponding to the slope of the ground that are used to control the posture are described in this section.

### 4.1. Leg Length Control for Adaptation to Terrain Elevation in the Double Support Phase

**Figure 12:**
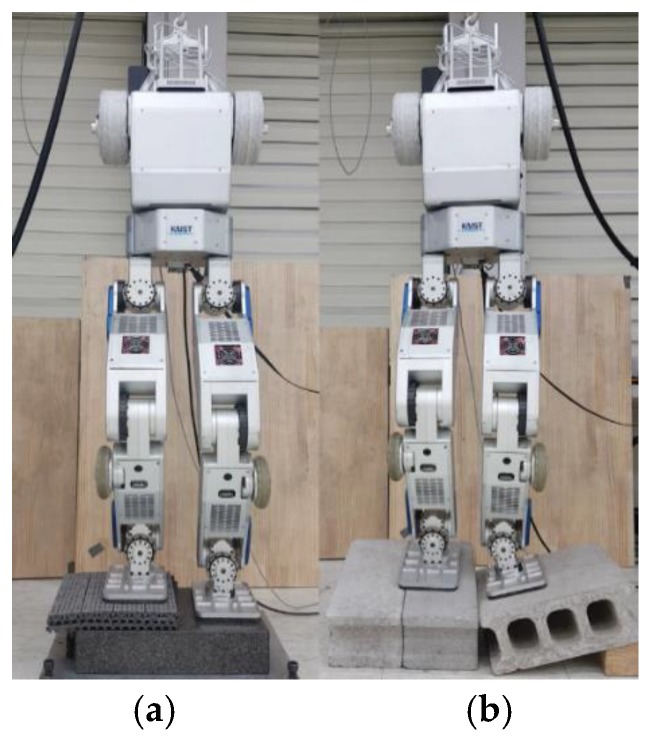
Leg length and foot orientation control on uneven terrain. (**a**) Height difference and (**b**) both height and orientation difference.

If the height of the floor varies, as shown in Figure 12a, the leg length of the robot should be adjusted to maintain desired posture. In [20], the desired ZMP is generated from linear inverted pendulum tracking, and a force reference is generated to adjust the vertical leg length. In a similar approach, we generate the force reference that each foot of the robot should apply on the floor using reference $z_{cp}$ generated through a capture-point control, as follows:

(8) $$f_{L}^{d}=k_{f}Mg,\;\;f_{R}^{d}=(1-k_{f})Mg,\;k_{f}=\frac{\left|z_{cp,Y}-P_{Y,L}\right|}{\left|P_{Y,L}-P_{Y,R}\right|}\;(0\leq k_{f}\leq 1),$$

where subscripts R and L indicate the right and left legs, respectively, M is the mass of the robot, $P_{Y,L}$ and $P_{Y,R}$ are the positions of the left and right feet along the *y* direction, respectively, and $k_{f}$ is the gain distributing the force to each foot. Note that $z_{cp}$ determines the foot that should exert more force on the floor. Equation (8) was derived from [20].

**Figure 13:**
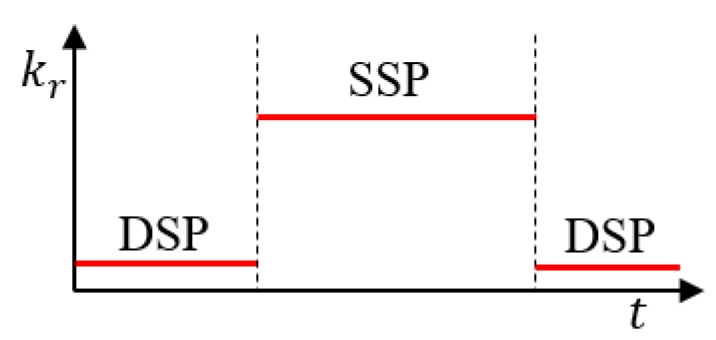
Gain scheduling according to the supporting phase. By increasing the gain $k_{r}$ in the single support phase, the modified leg length in double support phase returns to its original leg length in single support phase.

Using calculated force references $f_{L}^{d}$ and$\;f_{R}^{d}$ and measured vertical forces $f_{L}$ and $f_{R}$ retrieved from force/torque sensors on the feet, the leg length can be adjusted via the following PD control:

(9) $${\dot{u}}_{z}=k_{p}\left(\left(\Delta F\right)-\left(\Delta F^{d}\right)\right)+k_{d}\left(\left(\dot{\Delta F}\right)-\left({\dot{\Delta F}}^{d}\right)\right)-k_{r}u_{z},$$

where $k_{p}$ and$\;k_{d}$ are the proportional and derivative gains, respectively, with $k_{p}$, $k_{d}$ > 0; and $k_{r}$ is the gain that returns the control input ${\dot{u}}_{z}$ to zero. We experimentally tuned the proportional and derivative gains. Under the assumption that the floor is flat, $k_{r}$ is adjusted to retrieve the leg length, which is changed by the control input, back to the original length. Specifically, by scheduling the gain $k_{r}$ according to the support phase, as shown in Figure 13, the leg length in the double support phase (DSP) is adjusted because the gain $k_{r}$ is set to small and the adjusted leg length in the DSP returns to the original leg length in the single support phase (SSP) because the gain $k_{r}$ is set to large. In addition, if the elevation of the ground is constant, we can set $k_{r}$ in the SSP to be small enough to keep the previous control input. The calculated velocity input,$\;{\dot{u}}_{z}$, is integrated into position $u_{z}$ and added to the vertical position of the right and left feet as 0.5$\;u_{z}$ and −0.5$\;u_{z}$, respectively.

**Figure 14:**
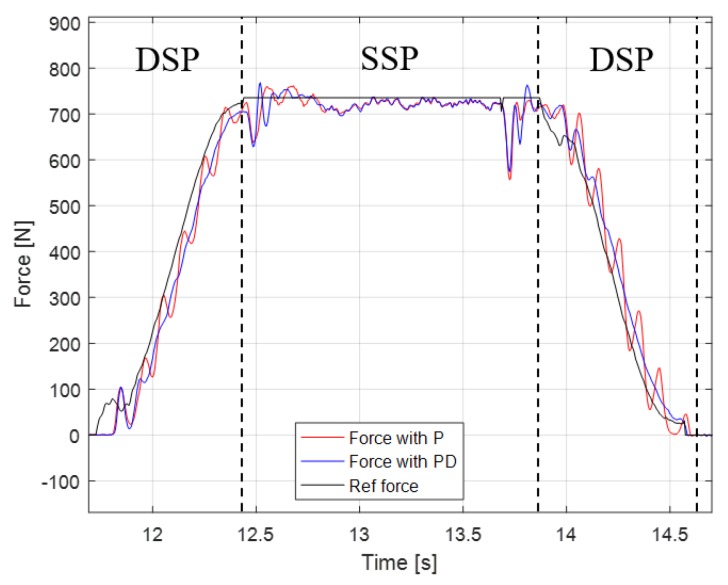
Leg length controller with proportional and proportional-derivative (PD) control.

In [20], only proportional gain is used, but increasing the proportional gain to improve the controller performance generates oscillation. By adding derivative gain, the resulting damping effect mitigates oscillation, while allowing a high proportional gain. Figure 14 shows the reference and measured forces of the right foot with and without the derivative gain at fixed proportional gain. Note that when the PD gain is applied, oscillation of the force is significantly diminished in the DSP. That is, a high P gain is applied for the fast adaptation of the leg length to the height difference of the floor in the DSP, and the vibration due to the high P gain is attenuated by the D gain. As mentioned earlier, in the SSP, the damping effect does not appear, because the third term of Equation (9), which retrieves the adjusted leg length relative to the original leg length, is dominant. Because the leg length is already adapted to the height difference in the DSP, the damping effect is not significant in the SSP.

### 4.2. Foot Orientation Control for Adaptation to Ground Slope

Besides the leg length, the foot orientation must be controlled to maintain the desired robot attitude, as shown in Figure 12b. Humans generate the desired torque on the foot according to the current state and adapt to the shape of the floor. Similarly, we calculate the desired torque of the robot foot from the difference between the reference and adjusted ZMPs, and the foot orientation is adjusted to track the desired toque.

The torque reference for the *y* axis of the right foot is the same as in Equation (9), and torques $\tau_{R,x}^{d}$,$\;\tau_{L,y}^{d}$, and $\tau_{L,x}^{d}$ are calculated analogously.

(10) $$\tau_{R,y}^{d}=f_{R}^{d}\left(z_{cp,x}-z_{ref,x}\right)=f_{R}^{d}\left(\Delta \xi_{x}\right),\;f_{L}^{d}=k_{t}Mg,\;f_{R}^{d}=(1-k_{t})Mg,\;k_{t}=\frac{\left|z_{ref,y}-P_{y,L}\right|}{\left|P_{y,L}-P_{y,R}\right|}\;(0\leq k_{t}\leq 1).$$

The desired torque is tracked by the following PD control:

(11) $${\dot{u}}_{ori}=k_{p}\left(\left(\tau^{d}-\tau \right)\right)+\;k_{d}\left(\left({\dot{\tau }}^{d}-\dot{\tau }\right)\right)-k_{r}u_{ori},$$

where $k_{p}$ and $k_{d}$ are the proportional and derivative gains, respectively, with $k_{p}$, $k_{d}$ > 0, and $\tau^{d}$ and τ are the desired and measured torques, respectively. Like for leg length control, the calculated velocity input ${\dot{u}}_{ori}$ is integrated into orientation $u_{ori}$ and added to the orientation of the corresponding foot.

## 5. Experiments and Results

To test the proposed balance control framework, we conducted three experiments. Each experiment was performed on the DRC-HUBO+ with all of the balance controllers designed in Section 3 and Section 4 simultaneously applied. The first experiment consisted of the robot maintaining balance on a sloping wooden plank. The second and third experiments were terrain-blind walking on uneven terrain and a stony area without terrain information, respectively. Videos from the experiments can be seen in [24].

### 5.1. Hardware Implementation for Real-Time Control

The control cycle of the framework is 5 ms and runs on an Intel NUC5i7RYH embedded computer in the body of the robot; the operating system of the computer is Linux Ubuntu and the robot program’s development environment is PODO [15], which was developed by the KAIST HUBO Lab and guarantees hard real-time control. A ZMP reference is generated at the capture-point controller every 5 ms and the balance controller receives the reference and generates control inputs. The control input for the position of the CoM is calculated from the ZMP controller of the balance controller and the control inputs for the foot position in the *z* direction and the foot orientation in the *x* and *y* directions are calculated from the leg length and foot orientation controllers. Control inputs designed in the task space are converted to joint angle references through inverse kinematics. Each joint angle reference generated is transmitted to the motor driver of each joint at 200 Hz through the can, and the angle reference is tracked through the PD position control in each motor driver. Because the position controller has a control period of 1 kHz, the joint angle references at 200 Hz are interpolated to 1 kHz. As the robot is moved by the motors, the sensor data from the gyro and F/T sensors are transmitted to the embedded PC every 5 ms. The control framework was programmed in the C ++ language.

### 5.2. Balance Control under Varying Slope

**Figure 15:**
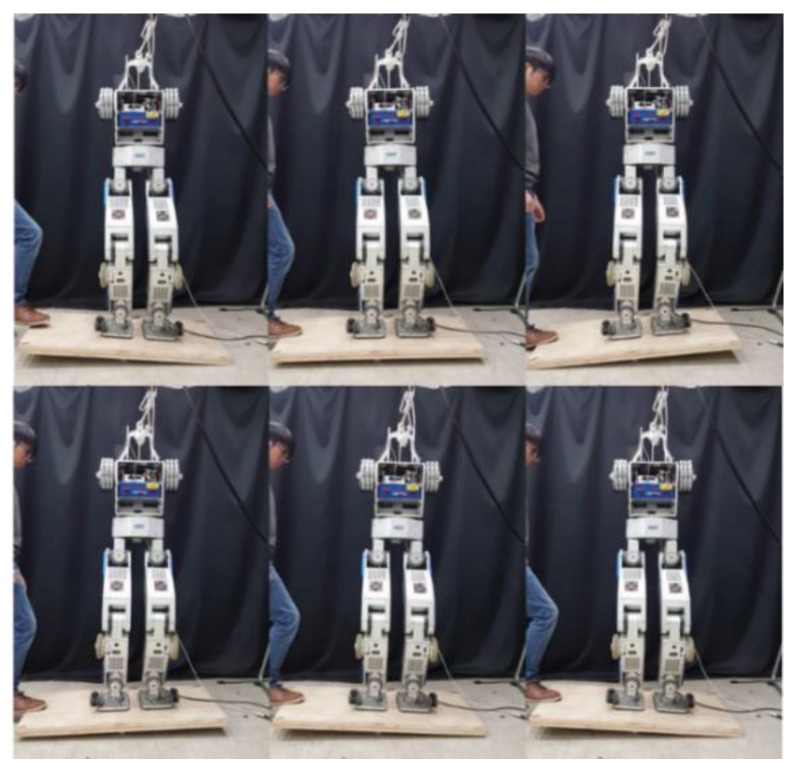
Snapshots of balance control experiment under varying inclination.

**Figure 16:**
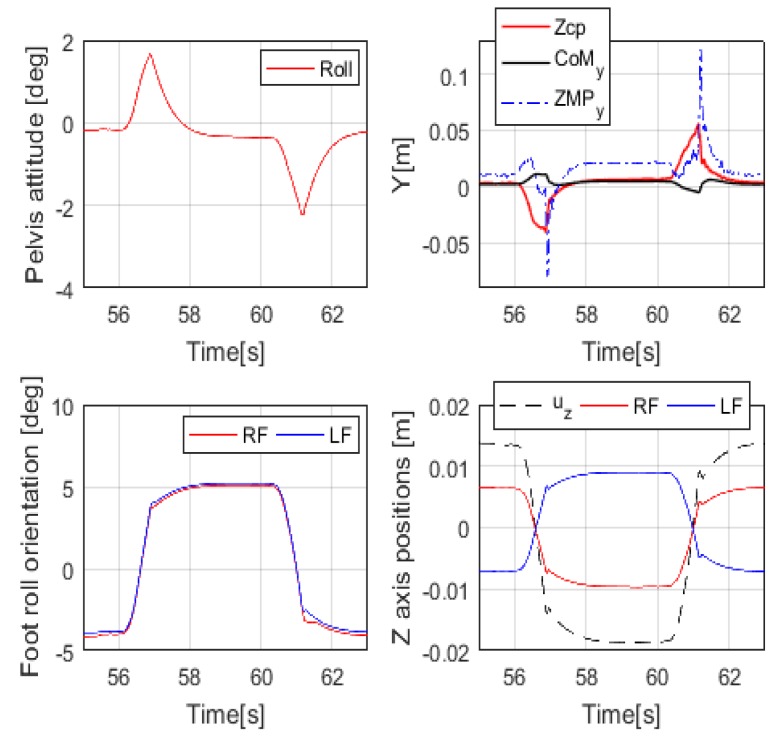
Experimental results from the robot on a varying incline.

Figure 15 shows the robot placed on a wooden board with a slope varying between 5° and –5°. We evaluated the performance of the proposed balance controller while arbitrarily modifying the slope. Figure 16 shows the experimental results corresponding to Figure 15 and slanting the wooden plank from 5° to –5° in the roll direction. As shown in the top-left graph in Figure 16, when a person tilts a wooden board at 5° (between 56 s and 59 s), the robot tilts in the plus roll direction (between 56 s and 59 s). At this moment, the $z_{cp}$ is adjusted through the capture-point control to maintain the balance of the robot, as shown in the upper-right graph of Figure 16. The adjusted $z_{cp}$ is used for the ZMP controller, and the ZMP controller adjusts the y-direction CoM (CoMy) to keep y-direction ZMP (ZMPy) at zero. Simultaneously with the ZMP control, $z_{cp}$ is input to the foot orientation controller and the leg length controller, and the foot orientation and leg length are adjusted to maintain the robot’s attitude at zero. The foot controller adjusted the direction of both feet by 5° in the roll direction as the floor tilted (shown in the bottom-right graph in Figure 16), and the leg length controller adjusted the right foot and left foot positions in the vertical direction by 0.5$u_{z}$ and –0.5$u_{z}$, respectively (shown in the bottom-left graph in Figure 16). When the slope in opposite direction, we can see that the balance controller operates on the same principle.

### 5.3. Terrain-Blind Walking on Uneven Terrain

**Figure 17:**
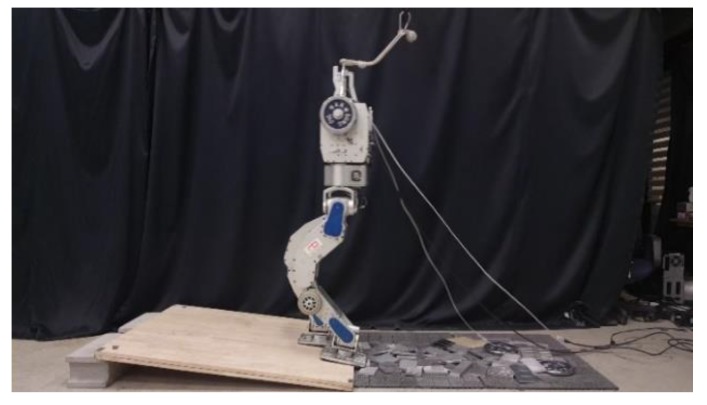
Snapshot of walking experiment on uneven terrain with varying local and global slopes.

In this experiment, terrain-blind walking tests were performed on uneven terrain, as shown in Figure 17. The uneven terrain consisted of a first section with a local slope and a second section with a global slope. The robot was commanded to walk on the local slope and the global slope. In the first section, aluminum and plastic plates with an area smaller than the foot sole area (24 cm × 16 cm) and a height of between 1 cm and 2 cm were placed randomly, and the second section had a slope of 8°. The robot walked with a stride of 0.15 m and a stepping cycle of 1 s (single support phase of 0.8 s and double support phase of 0.2 s).

**Figure 18:**
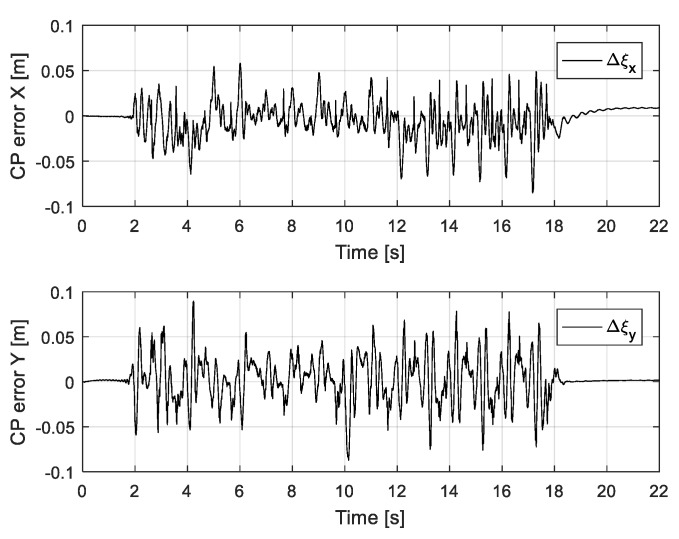
Measured capture-point error along x and y directions.

**Figure 19:**
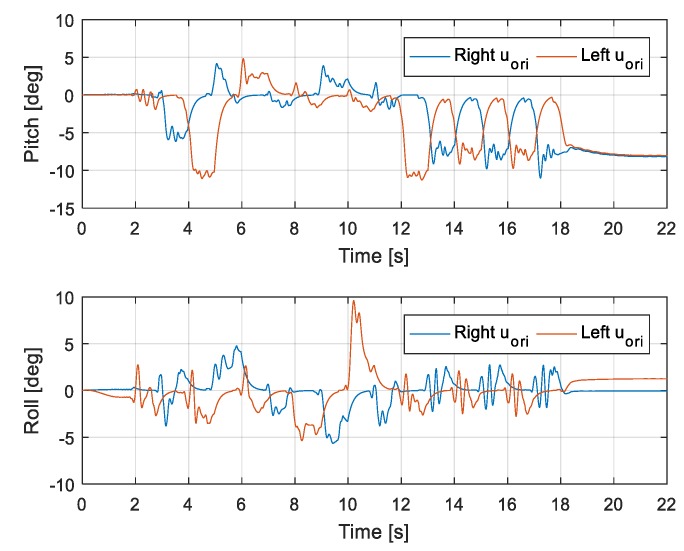
Foot orientation along x (roll) and y (pitch) axes.

**Figure 20:**
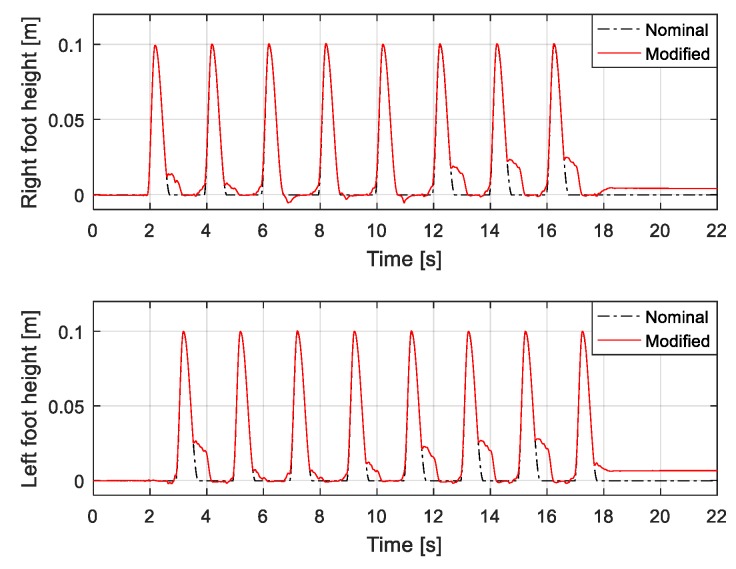
Feet height trajectory under varying local and global slopes.

**Figure 21:**
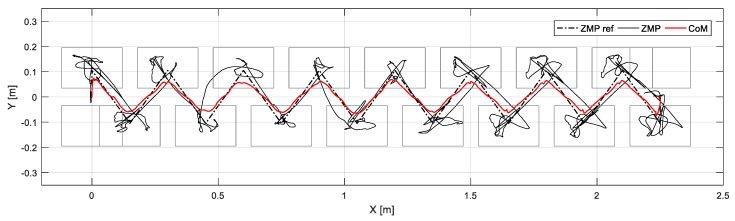
ZMP reference, measured ZMP, and CoM trajectory under varying local and global slopes.

Figure 18 shows capture-point error Δξ measured along the *x* and *y* directions, from when the robot walked on the uneven terrain in this experiment. The error variation with respect to the x-direction is relatively large because the inclination of the pitch direction is large when the robot walks on the global slope surface (from 12 s). Figure 19 shows the foot orientation input pitch changing to 8° after 12 s, almost agreeing with the global slope. In the experiment, we set a small time-constant *T* for the swinging foot, such that its orientation returned close to 0°. Figure 20 shows the nominal height trajectory (blue dot line in the figure) and modified height trajectory (red line in the figure) of both feet. The nominal foot height trajectory is modified by leg-length control. As the floor is higher than the expected landing position on the global slope, the foot position is high. Figure 21 shows the ZMP reference, measured ZMP and CoM trajectory. The grey rectangles in the graph represent the footprints. Because the front edge of each foot landed first on the slope when the robot was walking on the global slope, the change of ZMP was relatively large. However, the robot can stably walk on uneven terrain because the ZMP was maintained well within the boundary of foot by the balance controller.

### 5.4. Terrain-Blind Walking on a Stony Area and a Lawn

**Figure 22:**
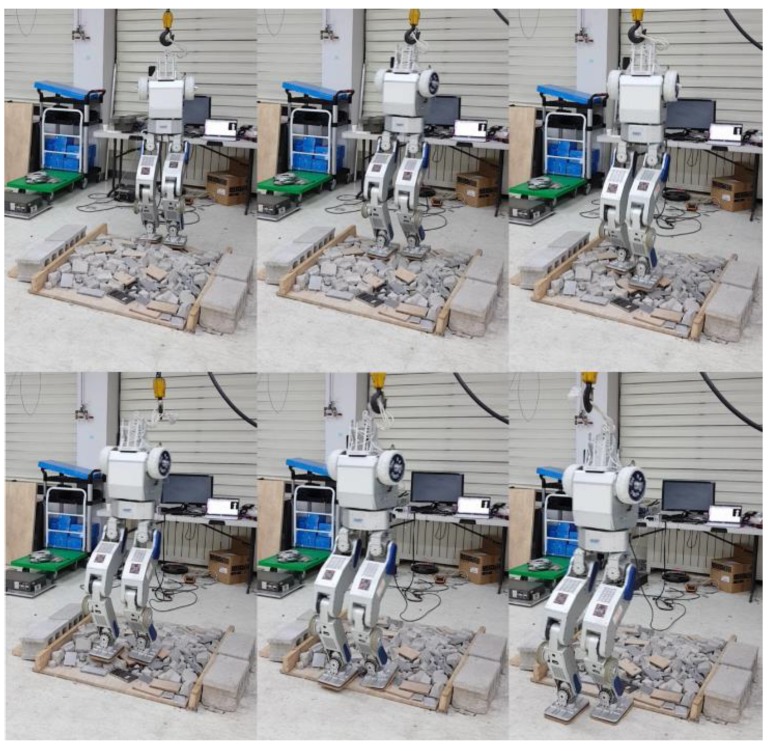
Snapshots from the walking experiment on a stony area.

**Figure 23:**
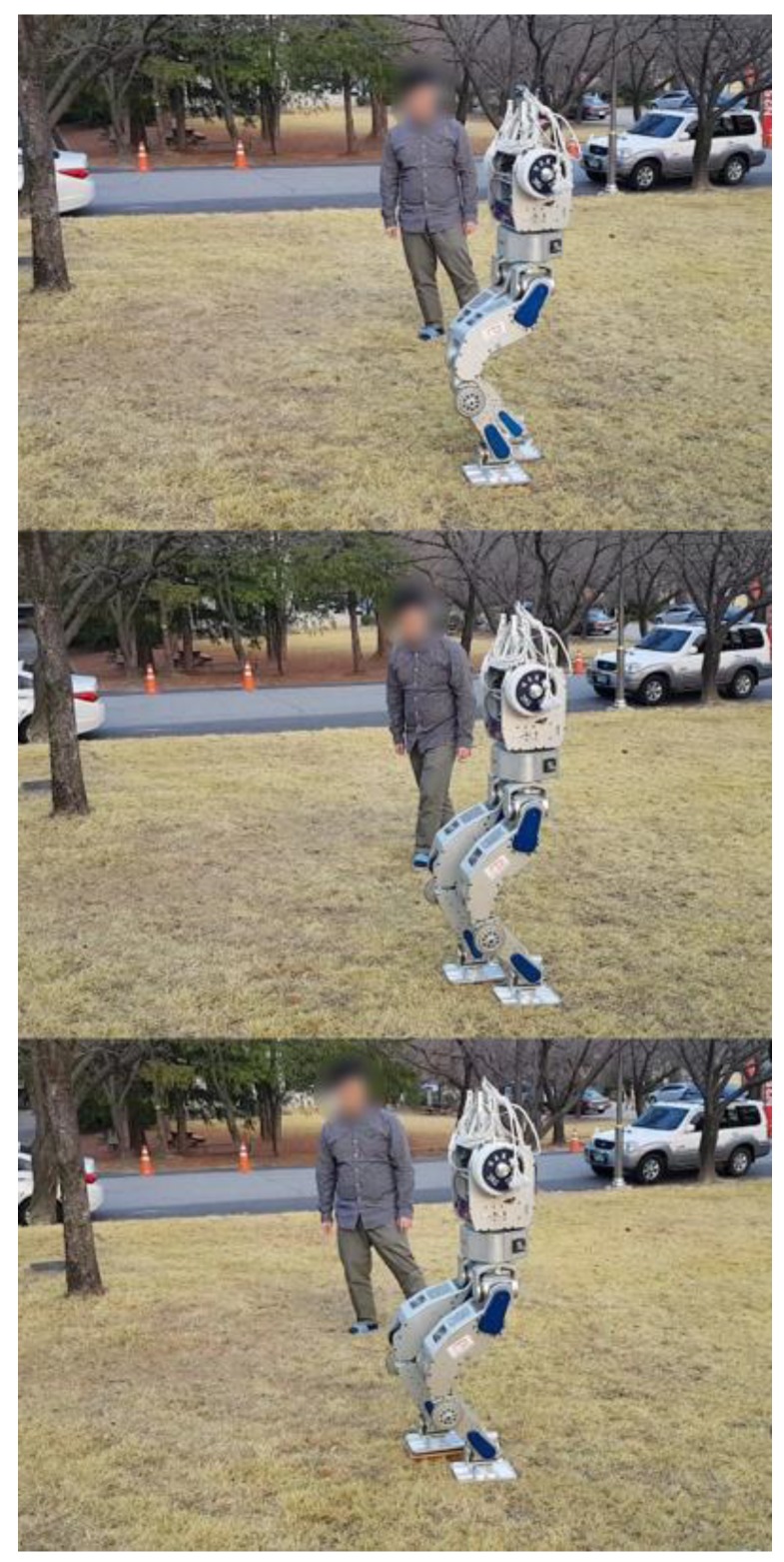
Snapshots from the walking experiment on a lawn.

To test the performance of the proposed balance control in a realistic environment, we conducted a terrain-blind walking experiment on a stony area and a lawn, as shown in Figure 22 and Figure 23. The stones were 5–10 cm width-wise, 5–10 cm in depth, and 2 cm to 4 cm in height. The robot walked with a stride of 0.15 m and a stepping cycle of 1 s (single support phase of 0.8 s and double support phase of 0.2 s). As stones move when the robot walks over them, a quick posture stabilization should be accomplished. With the proposed balance control framework, the robot was able to stably walk on the stony area. Furthermore, we also tested the robot walking on lawn. The lawn was relatively compliant, unlike the stony areas, but this had no significant impact on walking performance, and the robot walked stably on the lawn. Related experimental videos can be seen in [29].

## 6. Conclusions and Future Work

To realize the stable walking of a humanoid robot despite disturbances caused by unknown and uneven terrain, we designed a balance-control framework based on the capture-point control. The proposed framework consists of a capture-point controller and a balance controller that tracks the generated reference. The reference of each balance controller is derived from the desired ZMP, which is in turn generated by capture-point control. By using that control approach, the horizontal CoM and desired posture are stabilized on uneven terrain. The proposed control framework enabled the modified DRC-HUBO+ to stably walk on varying local/global slopes, a stony area, and lawn without terrain information. Moreover, this control framework can be easily implemented on other humanoid robots to improve walking performance, given its simplicity and robustness, guaranteed by the capture-point control.

We consider that the control robustness can be further improved in terms of walking performance if combined with foot-placement control. Hence, we will include a walking pattern with foot placement control into the proposed balance control framework in a future development. Furthermore, the authors believe it will contribute to more robust walking when combined with a terrain sensing algorithm in the future.

## Figures and Tables

**Table 1:** Specification of DRC-HUBO+.

Item		Description
Height		170 [cm]
Weight		80 [kg]
Degree of freedom (DOF)	Total	32 DOFs
	Arm	2 Arms × 7 DOFs
	Hand	2 Hands × 1 DOF
	Waist	1 DOF
	Leg	2 Legs × 6 DOFs
	Wheel	2 Wheels × 1 DOF
	Head	1 DOF
Actuators		200 [W], 100 [W] BLDC/DC Motor
Sensors		3-axis fiber-optics gyro, 3-axis inertia measurement unit (IMU)
Power		DC voltage: 48 [V], Capacity: 11.4 [Ah]

## Supplementary Materials

Experiment videos are available at https://youtu.be/4xk-UP4Qcfo.
